# The complex role of Wnt ligands in type 2 diabetes mellitus and related complications

**DOI:** 10.1111/jcmm.16663

**Published:** 2021-05-27

**Authors:** Xiaobo Nie, Xiaoyun Wei, Han Ma, Lili Fan, Wei‐Dong Chen

**Affiliations:** ^1^ Key Laboratory of Receptors‐Mediated Gene Regulation and Drug Discovery School of Basic Medical Sciences People’s Hospital of Hebi Henan University Kaifeng China; ^2^ Key Laboratory of Molecular Pathology School of Basic Medical Science Inner Mongolia Medical University Hohhot China

**Keywords:** canonical Wnt signalling pathway, complications, diabetes, non‐canonical Wnt signalling pathway, Wnt‐based therapeutics

## Abstract

Type 2 diabetes mellitus (T2DM) is one of the major chronic diseases, whose prevalence is increasing dramatically worldwide and can lead to a range of serious complications. Wnt ligands (Wnts) and their activating Wnt signalling pathways are closely involved in the regulation of various processes that are important for the occurrence and progression of T2DM and related complications. However, our understanding of their roles in these diseases is quite rudimentary due to the numerous family members of Wnts and conflicting effects via activating the canonical and/or non‐canonical Wnt signalling pathways. In this review, we summarize the current findings on the expression pattern and exact role of each human Wnt in T2DM and related complications, including Wnt1, Wnt2, Wnt2b, Wnt3, Wnt3a, Wnt4, Wnt5a, Wnt5b, Wnt6, Wnt7a, Wnt7b, Wnt8a, Wnt8b, Wnt9a, Wnt9b, Wnt10a, Wnt10b, Wnt11 and Wnt16. Moreover, the role of main antagonists (sFRPs and WIF‐1) and coreceptor (LRP6) of Wnts in T2DM and related complications and main challenges in designing Wnt‐based therapeutic approaches for these diseases are discussed. We hope a deep understanding of the mechanistic links between Wnt signalling pathways and diabetic‐related diseases will ultimately result in a better management of these diseases.

## 1 INTRODUCTION

Diabetes, caused by the deficiency of insulin secretion and/or insulin resistance and characterized by chronic hyperglycaemia, is currently one of the most important metabolic diseases worldwide. Diabetic patients have a higher risk of developing a series of acute metabolic complications, such as diabetic ketoacidosis, and chronic vascular complications (angiopathy) including microvascular diseases such as diabetic retinopathy (DR), diabetic peripheral neuropathy (DPN), diabetic nephropathy (DN) and diabetic foot, and macrovascular diseases including cardiovascular disease manifesting as myocardial infarction and cerebrovascular disease resulting in strokes.^1^, ^2^ The prevalence and incidence rate are increasing rapidly in most countries, according to the latest data from International Diabetes Federation, more than 463 million adults are suffering from diabetes and the number is expected to rise to 700 million by 2045. Diabetes caused 4.2 million deaths and 10% of total health expenditure on adults in 2019. More severely, about 50% people with diabetes have not been diagnosed and about 79% of adults with diabetes are living in developing countries. Diabetes is mainly divided into type 1 diabetes mellitus (T1DM), type 2 diabetes mellitus (T2DM) and gestational diabetes mellitus (GDM); among them T2DM is the most common type and accounts for above 90% of all diabetes cases.^3^ It is commonly believed that insulin resistance is the initial factor for the occurrence of T2DM, whereas dysfunction of pancreatic β‐cells is the determinant factor.^4^, ^5^ There is no cure for T2DM currently, the cornerstone of the treatment is reducing insulin resistance and stimulating pancreas to secret more insulin. Therefore, it is urgent to reveal the underlying pathogenic mechanisms of T2DM for a better therapeutic management.

The aetiology of T2DM has not been fully elucidated, and it is considered to be a complex polygenetic disease attributed to the interaction between hereditary predisposition and multiple acquired disposition; the latter of which includes the risk factors such as overweight, unhealthy diet, physical inactivity, increasing age and hypertension.^6^, ^7^ The abnormalities in many important signalling transduction pathways are critically involved in the occurrence and progression of T2DM and related complications.^8^, ^9^, ^10^, ^11^ Among them Wnt signalling pathways attract more attention due to the essential role in the embryogenesis and tissue homeostasis, and notorious role in the pathogenesis of multiple human diseases, especially in cancers.^12^, ^13^, ^14^ The relationship between Wnt signalling pathways and T2DM was firstly documented by Grant *et al* in 2006; they found genetic polymorphism of *TCF7L2* gene, which encodes an important transcription factor TCF4 in Wnt signalling pathways, contributed to the risk of T2DM through regulation of the expression of proglucagon gene.^15^ Subsequently, emerging studies proved that dysregulation of Wnt signalling pathways participate in the occurrence and progression of T2DM through directly influencing the differentiation and proliferation of pancreatic β‐cells and the secretion and action of insulin.^16^, ^17^, ^18^ However, due to the numerous components and resulting intricate networks, the role of Wnt pathways in the pathogenesis of T2DM and related complications seems to be contradictory, sometimes they function as protectors, while their activation is simultaneously required for the development of these disorders, and our understanding on their relationship is still quite rudimentary. Therefore, a more comprehensive understanding of their relationship will be helpful for a better therapeutic effect.

## 2 THE CANONICAL AND NON‐CANONICAL WNT SIGNALLING PATHWAYS

**FIGURE 1:**
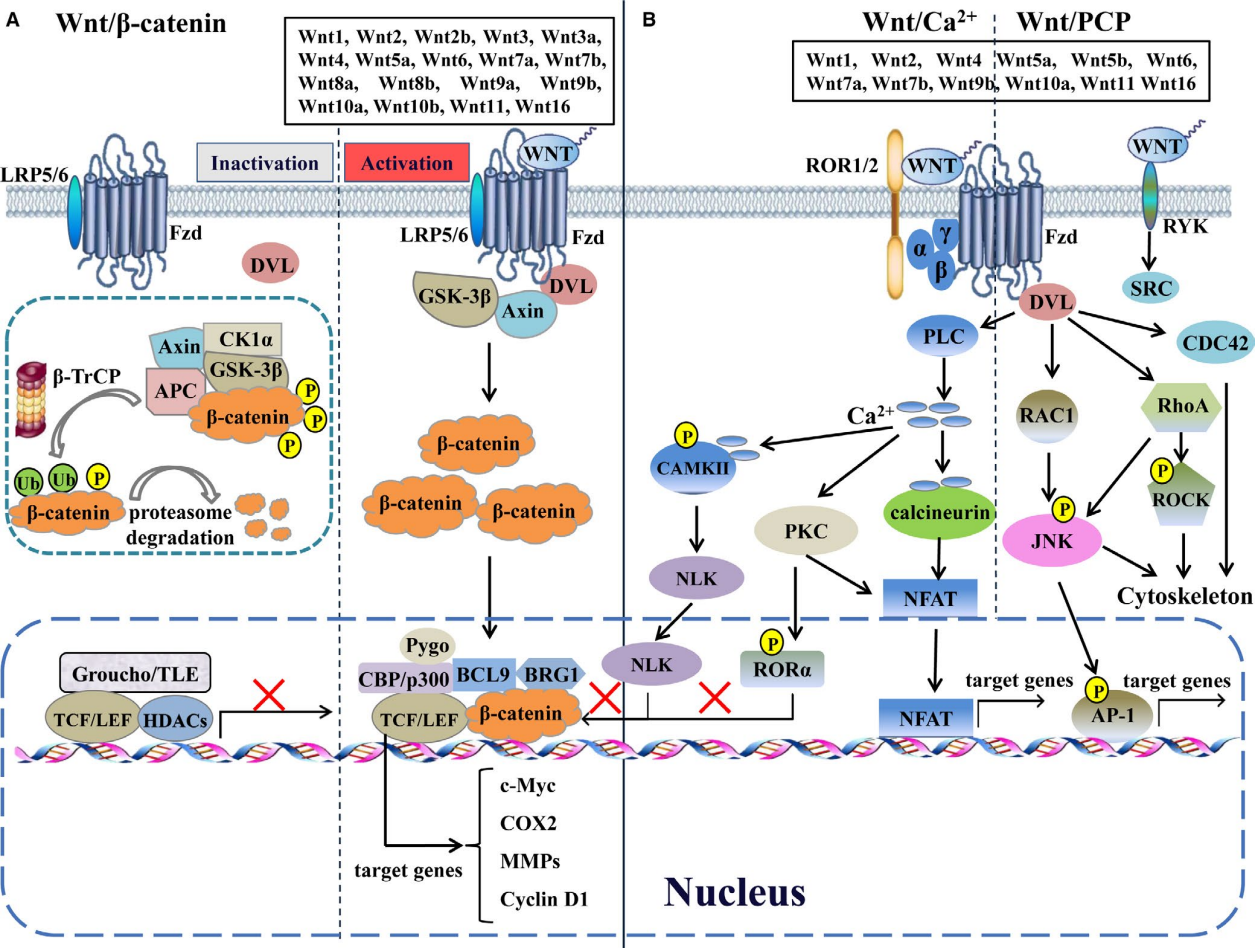
Multiple Wnt signalling pathways. Various Wnts regulate the canonical Wnt/β‐catenin pathway, the non‐canonical Wnt/planar cell polarity (PCP) pathway and Wnt/Ca^2+^ pathway. AP‐1, activator protein 1; APC, adenomatosis polyposis coli; BCL9, B cell CLL/lymphoma 9; BRG1, Brahma‐related gene 1; β‐TRCP, β‐transducin repeat‐containing protein; CAMKII, calmodulin‐dependent protein kinase II; CDC42, cell division cycle 42; CK1α, casein kinase 1; COX2, cytochrome c oxidase subunit 2; DVL, dishevelled; Fzd, frizzled; GSK‐3β, glycogen synthase kinase; HDACs, histone deacetylases; JNK, JUN N‐terminal kinase; LEF, lymphoid enhancer–binding factor; LRP5/6, low‐density lipoprotein receptor–related protein 5/6; MMPs, matrix metalloproteinases; NFAT, nuclear factor of activated T cells; NLK, nemo‐like kinase; PKC, protein kinase C; PLC, phospholipase C; RAC1, Rac family small GTPase 1; ROCK, Rho kinase; ROR1/2, receptor tyrosine kinase–like orphan receptor 1/2; RORα, retinoic acid–related orphan nuclear receptor α; RYK, receptor like tyrosine kinase; TCF, T cell factor; TLE, transducin‐like enhancer protein

The Wnt signalling pathway is roughly divided into β‐catenin‐dependent (canonical) and β‐catenin‐independent (non‐canonical) signalling pathways, which activates distinct intracellular signalling pathways (Figure 1). Among them the canonical Wnt signalling gets more attention and is well understood.^19^ The most crucial event in this signalling is the regulation in the turnover of β‐catenin, a pivotal component that acts as a transcriptional co‐activator in this cascade. In resting cells, the production of Wnts is suppressed and the protein level of cytoplasmic β‐catenin is low due to the activity of destruction complex composed of Axin, adenomatosis polyposis coli (APC), casein kinase 1 (CK1α) and glycogen synthase kinase (GSK‐3β). β‐catenin is captured and phosphorylated by the destruction complex,^20^ followed by ubiquitinated by β‐transducin repeat‐containing protein (β‐TRCP) and dispatched to the proteasome for complete degradation,^21^ without enough β‐catenin in nucleus, the bidirectional T cell factor/lymphoid enhancer–binding factors (TCF/LEF) begin to recruit transducin‐like enhancer protein (Groucho/TLE) and histone deacetylases (HDACs) to form a repressive complex, thus to inhibit the transcription of Wnt target genes. Conversely, the activation of canonical Wnt signalling is initiated by the formation of complex among Wnts, their cognate receptor Frizzled (Fzd) and coreceptor low‐density lipoprotein receptor–related protein 5/6 (LRP5/6) on the cell membrane. Consequently, the effector protein dishevelled (DVL) is recruited and polymerized to inactivate the destruction complex, which leads to the stabilization and accumulation of β‐catenin in the cytoplasm and the subsequent translocation into the nucleus to form an active complex with TCF/LEF by removing TLE/Groucho complexes and recruiting transcriptional co‐activators such as B cell CLL/lymphoma 9 (BCL9), Brahma‐related gene 1 (BRG1), CBP/p300 and Pygo. Finally, the transcription of Wnt target genes is driven and results in the changes of series of cellular processes. Collectively, the activation of canonical Wnt signalling mainly includes the following biological processes: the production and secretion of Wnts, the recognition of Wnts by their receptors, the inactivation of destruction complex, the accumulation of β‐catenin and translocation into nucleus and the activation of transcriptional complex of target genes.

The non‐canonical Wnt signalling is mainly subdivided into Wnt/Ca^2+^ and Wnt/planar cell polarity (PCP) signalling pathways, which are activated by some Wnts proteins, such as Wnt5a, Wnt5b and Wnt11, and eventually regulate the cellular polarity and migration‐related signalling pathways that have important roles in cell orientation during development and cell migration in metastasis formation.^22^ The Wnt/Ca^2+^ signalling is activated by the complex formation among Wnt, Fzd, DVL and G proteins and the resulting activation of phospholipase C (PLC) activity and subsequent calcium influx, increased intracellular Ca^2+^ concentration activates various signalling pathways, including protein kinase C (PKC), Ca^2+^/calmodulin‐dependent protein kinase II (CAMKII) and Ca^2+^/calcineurin, leading to the phosphorylation of retinoic acid–related orphan nuclear receptor α (RORα) and/or the translocation of transcription factors, such as nuclear factor activated in T cells (NFAT) and Nemo‐like kinase (NLK).^23^ Intriguingly, RORα and NLK function as inhibitors of canonical Wnt signalling via reducing the binding of β‐catenin to TCF/LEF transcription factors.^24^, ^25^ In the Wnt/PCP signalling, Wnts bind to the receptor like tyrosine kinase (RYK) to active SRC, or to the receptor tyrosine kinase–like orphan receptor 1/2 (ROR1/2)‐Fzd complex to activate DVL and further activate small Rho GTPases, including Rac family small GTPase 1 (RAC1), RhoA and cell division cycle 42 (CDC42), in a DVL‐dependent way. RhoA triggers ROCK and c‐Jun N‐terminal kinase (JNK) but RAC1 only activates JNK, thereby regulating rearrangements of cytoskeleton and/or activating related transcription factors, such as activator protein 1 (AP‐1) and NFAT. Moreover, some new non‐canonical Wnt signalling pathways, including Wnt/STOP signalling, Wnt/TOR signalling, Wnt‐YAP/TAZ signalling, Wnt/LRP5/mTOR/Akt signalling and Wnt/Hippo signalling have been discovered gradually and their roles in physiology and pathology has been well explored and discussed.^26^, ^27^, ^28^, ^29^ Theoretically, changes in any component involving in Wnt signalling pathways may result in the abnormalities of these pathways, and several studies have elaborately summarized the impact of Wnt pathways on T2DM and related complications.^16^, ^17^, ^18^ In this review, we only focus on the current insights and recent advances in the role of each human Wnt and main related antagonists (sFRPs and WIF‐1) and coreceptor (LRP6) in the pathogenesis of T2DM and related complications.

## 3 ROLES OF WNTS IN T2DM AND RELATED COMPLICATIONS

As initiators of Wnt signalling pathways, Wnts are a cluster of conserved secreted glycoproteins and widely expressing in all metazoan species; humans carry 19 members of Wnts with 40%‐90% amino acid sequence identity. Among them Wnt2b, Wnt3, Wnt3a, Wnt8a, Wnt8b, Wnt9a and Wnt10b mainly activate the canonical pathway, Wnt5b and Wnt11 activate the non‐canonical pathway, and Wnt1, Wnt2, Wnt4 Wnt5a, Wnt6, Wnt7a, Wnt7b, Wnt9b, Wnt10a and Wnt16 function as initiators for both signalling pathways. The structural characteristics and the maturation of all human Wnts have been elaborately summarized by our group and Kikuchi et al.^19^, ^30^ However, the detailed mechanism by which single Wnt is chosen to produce and activate specific Wnt pathway has not been fully clarified. In the following parts, the exact role of each Wnt in the development of T2DM and related complications will be discussed separately (Table 1).

**TABLE 1:** Wnt activating the canonical and non‐canonical Wnt signalling pathways in the pathogenesis of diabetic‐related diseases

Wnts	Expression Pattern in diabetic samples	Effect on diabetic‐related diseases		Type of Wnt signalling	Reference
Wnt1	No data	Deleterious	Diabetic nephropathy	Canonical	31
		Protective	Diabetic microvascular disease	Canonical	32
Wnt2	Increased in myocardial tissues	Deleterious	Diabetic cardiomyopathy	Canonical	33
	Increased in retinal endothelial cells	Deleterious	Diabetic retinopathy	No data	34
Wnt2b	Increased in islets	Deleterious	Dysfunction of islet	Canonical	36
	Increased in serum	Deleterious	Diabetic nephropathy	Canonical	37, 38
Wnt3	Decreased in olfactory bulbs and hippocampus	Protective	Diabetic central nervous system complication	Canonical	41, 42
Wnt3a	Decreased in cardiomyocytes	Protective	Diabetic cardiomyopathy	Canonical	43
	Increased in gastrocnemius	Deleterious	Insulin resistance	Canonical	44
	No data	Protective	Impaired proliferation of β‐cells and insulin secretion	Canonical	47, 48
	No data	Protective	Impaired bone formation	Canonical	49
	Increased in kidney	Deleterious	Diabetic nephropathy	Canonical	50
	Increased in vitreous fluid	Deleterious	Diabetic retinopathy	No data	51
	Increased in aortic tissues	Deleterious	Diabetic macrovascular disease	No data	52
	Decreased in adipose tissues	Protective	Adipogenesis and obesity	Canonical	53, 54
Wnt4	Decreased in pre‐diabetic but increased in diabetic in islets	Deleterious	Impaired proliferation of β‐cells and insulin secretion	Non‐canonical	59, 60
	Decreased in islets	Protective	Apoptosis and dysfunction of islet	Canonical	61
	Decreased in kidney	Protective	Diabetic nephropathy	Canonical	62
	Increased in kidney	Deleterious	Diabetic nephropathy	Canonical	67, 68
	Increased in carotid arteries	Deleterious	Diabetic macrovascular disease	Canonical	69
Wnt5a	Increased in islets	Deleterious	Apoptosis and dysfunction of islet	Canonical/ Non‐canonical	61, 82
	Decreased in islet stellate cells	Protective	Impaired insulin secretion	Canonical	70, 84
	Increased in adipose tissues	Deleterious	Impaired insulin secretion	Non‐canonical	78
	Decreased in kidney	Protective	Diabetic nephropathy	Canonical	62
	Increased in plasma	Deleterious	Insulin resistance	Non‐canonical	75, 76
	Increased in peripheral blood macrophages	Deleterious	Diabetic macrovascular disease	Non‐canonical	87, 88
	Increased in ischaemic muscles and endothelial cells	Deleterious	Diabetic foot ulcers	Non‐canonical	92
	Increased in plasma, decreased in glomerular mesangium	Protective	Diabetic nephropathy	Canonical	95, 96
Wnt5b	Increased in adipose tissues	Deleterious	Adipogenesis and obesity	Non‐canonical	99, 100
Wnt6	Decreased in adipose tissues	Protective	Adipogenesis and obesity	Canonical	101, 102
	Decreased in kidney	Protective	Diabetic nephropathy	Canonical	103
Wnt7a	Increased in aortic tissues	Deleterious	Diabetic macrovascular disease	Canonical	52, 108, 111
	Decreased in wounded skin tissues	Protective	Diabetic wounds	Canonical	108
	Decreased in hippocampus	Protective	Diabetic central nervous system complication	No data	109
Wnt7b	No data	Protective	Diabetic arteriosclerosis	No data	110
Wnt8a	No data				
Wnt8b	No data				
Wnt9a	No data	Deleterious	Impaired differentiation of β‐cells	Canonical	113
	No data	Protective	Impaired Insulin secretion	No data	114
Wnt9b	No data	Protective	Diabetic foot ulcers	Canonical	115
Wnt10a	Increased in brown pre‐adipocyte cells	Protective	Adipogenesis and obesity	Canonical	117, 118
	Increased in spinal cord tissues	Deleterious	Diabetic neuropathy	Canonical	119
Wnt10b	Decreased in white adipose and skeletal muscle tissues	Protective	Adipogenesis and obesity, insulin resistance	Canonical	120, 121, 122, 123, 124, 125
	Decreased in bone	Protective	Diabetic bone loss	Canonical	127
Wnt11	Increased in adipose tissues	Deleterious	Adipogenesis and obesity	Non‐canonical	129, 130
Wnt16	Decreased in cortical bone	Protective	Diabetic osteopenia	Canonical/Non‐canonical	132, 133
	Decreased in corpus cavernosum tissues	Protective	Diabetic erectile dysfunction	No data	134

### 3.1 Wnt1, Wnt2 and Wnt2b

At present, no direct evidence is available for the link between Wnt1 and T2DM; few studies on its role in different diabetic complications is also controversial. In T1DM‐induced rats, the inhibition of Wnt1/β‐catenin signalling by salidroside, a hypoglycaemic and antioxidant glycoside, was found to protect against DN, indicating a deleterious role of Wnt1 in diabetes.^31^ However, Chong *et al* showed that erythropoietin (EPO) could protect endothelial cells (ECs) against elevated glucose exposure through activating Wnt1/β‐catenin pathway, suggesting a protective role of Wnt1 against diabetic microvascular disease.^32^ Similarly, there are few studies concerning the role of Wnt2 in T2DM. An in vivo study shows that the mRNA and protein levels of Wnt2, β‐catenin and Wnt target genes are all increased progressively in myocardial tissues from rat following streptozotocin (STZ)‐induced diabetes, which is accompanied by the cardiac dysfunction and progressive cardiomyocyte apoptosis, suggesting that the activation of Wnt2/β‐catenin pathway may facilitate the development of diabetic cardiomyopathy.^33^ Furthermore, a recent study shows that circular RNA circHIPK3 promotes DR by increasing the expression of Wnt2 in retinal ECs.^34^ These data suggest that Wnt2 may be a potential target to control these diabetic complications. Wnt2b shares 70% amino acid sequence identity with Wnt2. Zhou *et al* found the epistasis between *Wnt2b* and *TCF7L2* genes was associated with the susceptibility of T2DM in Chinese Han population.^35^ The expression of Wnt2b and mediated canonical Wnt pathway is induced and inversely correlated with insulin expression in islets of T2DM patients, indicating inhibition of this pathway might be a new route to prevent the failure of β‐cells.^36^ Moreover, Wnt2b is up‐regulated in serum of patients with DN and mesangial cells cultured in high glucose environment, and high glucose treatment could enhance the inflammation and extracellular matrix via activating the Wnt2b/β‐catenin pathway induced by two different long non‐coding RNAs, providing new mechanisms for understanding the development of DN and promising target for its treatment.^37^, ^38^

### 3.2 Wnt3 and Wnt3a

Wnt3 and Wnt3a share 85% amino acid sequence identity. At present, studies on Wnt3 are mainly focused on its role in malignancies rather than in diabetes; we have revealed the carcinogenic role of Wnt3 in the development of gastric and colorectal cancers,^39^, ^40^ and the current few studies on Wnt3 are mainly about its role in diabetic complication of central nervous system. A recent study revealed that the impaired cognitive function and loss of neurogenesis was associate with the inhibition of Wnt3/β‐catenin pathway in diabetic rat, and insulin treatment promoted neurogenesis via increasing the expression of Wnt3 in astrocytes.^41^ Similarly, the canonical Wnt3 pathway is inhibited in the hippocampus of diabetic rat, and treadmill exercise alleviated Alzheimer disease–associated memory loss in diabetic rats by increasing neurogenesis through activating this signalling.^42^ These results shed lights on the implementation of Wnt3 as a therapeutic candidate for targeting diabetic complication of central nervous system. Compared to its homologue, Wnt3a is known to regulate the pathogenesis of T2DM from several processes but its role is controversial. Under the similar hyperglycaemia condition in STZ‐induced diabetic rat, Wnt3a/β‐catenin pathway was found to be inhibited in cardiomyocytes,^43^ but swimming exercise was proved to alleviate insulin resistance through inhibiting the overactivated Wnt3a/β‐catenin pathway in skeletal muscles.^44^ Wnt3a is an important modulator of the differentiation and maturation of β‐cells, overexpressing Wnt3a promotes the proliferation of porcine pancreatic stem cells (PSCs), which are valuable in transplantation application of T2DM.^45^ Similarly, activation of the canonical Wnt pathway by treating exogenous Wnt3a enhances the differentiation of human embryonic stem cells (hESCs) to pancreatic lineage cells and induces the proliferation of β‐cells and insulin secretion in vitro.^46^, ^47^ However, in a latest study on human‐induced pluripotent stem cell (hiPSC)–derived S7 cells, activating the canonical Wnt3a/β‐catenin and non‐canonical Wnt4/5a/5b signalling pathways simultaneously did not alter or improve glucose‐simulated insulin secretion (GSIS); instead, inhibition of these endogenous Wnts even modestly promoted the maturation of β‐cells.^48^ In another study, activation of Wnt3a/β‐catenin pathway was found to improve the impaired osteointegration under diabetic condition.^49^ Wnt3a/β‐catenin signalling is also overactivated in the kidneys of diabetic patients and animal models, hence inhibiting this signalling by PPARα displays a protective effect on DN.^50^ Consistently, a significantly higher level of Wnt3a is detected in vitreous fluid samples from patients with proliferative DR compared with that in healthy subjects,^51^ and the levels of Wnt3a and Wnt7a are up‐regulated in aortic tissues from mice with diabetic macrovascular disease.^52^ Of note, Wnt3a also participates in the occurrence of T2DM by impacting adipocyte differentiation. Wnt3a and Wnt10b inhibits adipogenesis and related obesity via activating the canonical Wnt pathway.^53^, ^54^ Therefore, activation of canonical Wnt3a pathway has the effect of bidirectional regulation on T2DM and complications, depending on context, and further studies are needed to elucidate detailed mechanism of Wnt3a action in these disorders.

### 3.3 Wnt4

Wnt4 is the most abundantly expressed Wnt protein in β‐cells, whereas its role in diabetes is also contradicting because it functions as a biphasic initiator for canonical and non‐canonical Wnt pathways. Wnt4 is enriched in pancreatic islets of health mice and more abundant in insulin‐resistant obese mice, Wnt4 alone does not affect GSIS in primary murine β‐cells, but can antagonize the activation of canonical Wnt pathway mediated by Wnt3a and the resulting increase in cellular proliferation as well as GSIS in islets and β‐cell line INS1,^55^, ^56^ and depletion of Wnt4 results in a decrease in proliferation of INS1 cells.^57^ Similarly, cellular proliferation and GSIS of β‐cell line MIN6 are not affected when exposed to Wnt4 protein, but suppressed significantly when knockdown of Wnt4.^58^ Recently, Kozinski *et al* revealed the dynamic equilibrium of canonical and non‐canonical Wnt signalling pathways activated by Wnt3a and Wnt4 separately in β‐cells during the development of T2DM; they believed that in pre‐diabetic state, the increased secretion of Wnt3a and decreased secretion of Wnt4 by insulin‐resistant tissues were responsible for the increase in insulin secretion and β‐cell proliferation to adapt the systemic insulin resistance, whereas the expression profiles of Wnt3a and Wnt4 were reversed in a severe diabetic state, which correlated with the down‐regulated β‐cell proliferation and deficiency in insulin production.^59^ Wnt4 also induces the expression of transcriptional factors that are indispensable for islet differentiation such as NKX6.1 and PDX1 in human islets by activating the non‐canonical Wnt pathway.^60^ However, in another study, Wnt4 was found to reduce the protein levels of PDX1 and MAFA, and inhibiting Wnt4‐mediated non‐canonical signalling promoted the maturation of β‐cells.^48^ These opposite results might be attributed to the heterogeneity of islet, a mixture of different cell types that may dilute the effect of different transcriptional factors during the differentiation and maturation processes. In addition, Wnt4 was found to activate the canonical Wnt pathway in islet endothelium, and mesenchymal stromal cell (MSC)–based therapies ameliorated oxidative stress–induced apoptosis and functional impairment of islet endothelium via activating the canonical Wnt4 signalling.^61^ Moreover, Wnt4 is closely linked to the development of diabetic complications, especially in DN. High glucose condition induces transforming growth factor‐β1 (TGF‐β1)–mediated fibrosis in glomerular mesangial cells by inhibiting Wnt4‐ and Wnt5a‐mediated canonical pathways; therefore, restoring Wnt4 or Wnt5a significantly alleviates TGF‐β1‐mediated fibrosis in diabetic kidneys.^62^ Similarly, melatonin, simvastatin, Salvia miltiorrhiza extracts and liraglutide treatment block the apoptosis of mesangial cells and improve the renal injury of diabetic rat by restoring the canonical Wnt signalling mediated by Wnt4 and Wnt5a.^63^, ^64^, ^65^, ^66^ However, sitagliptin or soybean isoflavones treatment was reported to alleviate the renal tubulointerstitial fibrosis in rat with DN by inhibiting the canonical Wnt4 pathway.^67^, ^68^ Moreover, the canonical Wnt4 pathway is up‐regulated in carotid arteries of diabetic rats with carotid artery injury, and silencing this cascade by microRNA‐24 is sufficient to attenuate proliferation of vascular smooth muscle cells (VSMC) and neointimal hyperplasia.^69^ Overall, the role of Wnt4 varies depending on the tissue type; it mainly activates the non‐canonical Wnt pathway and acts as a negative regulator of canonical Wnt pathway in the context of islet, but mainly activates the canonical Wnt pathway in other tissues. In short, the general effect of Wnt4 seems to be deleterious, and further studies are required for a deeper understating of precise role of Wnt4 in diabetic‐related diseases.

### 3.4 Wnt5a and Wnt5b

Wnt5a, another initiator that activates the canonical and non‐canonical Wnt pathways, is closely related to a variety of metabolic disorders such as obesity and T2DM. Compared with that in healthy subject, plasma Wnt5a level is significantly decreased in patients with the onset T2DM, and a negative correlation is found between the Wnt5a level and fast blood glucose (FBG)/HbA1c levels. However, Wnt5a level is gradually increased in patients with long‐term T2DM or after 3 months of treatment,^70^, ^71^ and more studies demonstrate that the protein and mRNA levels of Wnt5a in circulation are elevated in obese individuals and patients with T2DM and positively correlated with IL‐6 concentration and metabolic disorders, such as increased triglyceride and FBG levels as well as insulin resistance.^72^, ^73^ Moreover, a mutual stimulation relationship between Wnt5a and inflammation cytokines is detected in cultured human adipocytes.^74^ Wnt5a is widely expressed in adipose tissues, and the deleterious role of Wnt5a through activating the non‐canonical Wnt pathway in the development of inflammation and insulin resistance has been clearly revealed.^18^, ^75^, ^76^ Recent studies showed that increased secretion of Wnt5a by enlarged adipocytes in the obese state could block insulin signalling and cause glucose intolerance via activating the non‐canonical Wnt5a/PCP pathway and systemic inflammation,^75^, ^77^ and an obviously positive correlation between the up‐regulated Wnt5a/PCP pathway and profound vascular insulin resistance were observed in visceral adipose tissue arterioles of obese individuals.^78^ Therefore, treatment of anti‐inflammatory cytokine IL‐10 in 3T3‐L1 pre‐adipocytes or celecoxib in diabetic rat could suppress the adipogenesis and reverse the non‐alcoholic steatohepatitis (NASH) via targeting this non‐canonical Wnt5a pathway.^79^, ^80^

Wnt5a is another regulator with dual function regarding the effect on islet function, induction of Wnt5a expression blocks glucose‐induced β‐cell proliferation,^81^ and treating β‐cells with exendin‐4, a glucagon‐like peptide‐1 receptor agonist regarded as a therapeutic reagent for T2DM, was found to promote β‐cell proliferation via inhibiting the expression of Wnt5a and mediated canonical Wnt pathway.^82^ However, in diabetic mice, the levels of Wnt5a and its receptor Fzd5 are significantly decreased in islet stellate cells (ISCs), a type of stellate cell located in islet and activated in T2DM, and down‐regulation of Wnt5a expression in ISCs could decrease insulin secretion,^83^ demonstrating a protective role of Wnt5a in maintaining the insulin secretion homeostasis of β‐cells. Similarly, Kuljanin *et al* found that the glucose‐lowering and islet regenerative capacities of bone marrow (BM)–derived multipotent stromal cells (MSCs) after transplantation into diabetic mice were mainly attributed to the activation of canonical Wnt5a pathway.^84^

Wnt5a is essential for normal development of heart, and increased oxidative stress in diabetic pregnancies causes heart defects in foetuses by inhibiting the expression of Wnt5a and its induced canonical and non‐canonical Wnt pathways; hence, overexpressing superoxide dismutase 1 (SOD1) in vivo could ameliorate heart defect through restoration of non‐canonical Wnt5a/Ca^2+^ pathway.^85^ However, the concentration of Wnt5a is elevated in the plasma and epicardial adipose tissue of patients with coronary artery disease (CAD) and independently associated with the presence of CAD and progression of calcified coronary plaque,^86^ and adipose tissue–derived Wnt5a in obesity induces arterial oxidative stress and migration of vascular smooth muscle cells (VSMCs) via activating a new Wnt5a/USP17/RAC1/NADPH oxidases axis.^87^ Moreover, oxidized low‐density lipoprotein (oxLDL)–induced activation of non‐canonical Wnt5a pathway and inhibition of canonical Wnt3a pathway could promote foam cell formation in human aortic VSMCs but inhibit their migration and proliferation.^88^ Overactivation of Wnt5a/PCP pathway is also detected in ECs from patients with T2DM, thus inhibiting this signalling could ameliorate insulin resistance and dysfunction of ECs from T2DM patients.^78^, ^89^ Wnt5a is also highly expressed in circulating monocytes and macrophages in patients with peripheral artery disease and in ischaemic muscle of ob/ob mice or mice fed with high‐fat and high‐sucrose diet. Myeloid‐specific Wnt5a overexpression blunts regenerative angiogenesis in ischaemic hind limbs by activating the non‐canonical Wnt5a/JNK pathway and mediated elevation of VEGF‐A165b, an antiangiogenic VEGF‐A splice isoform^90^; therefore, treatment with recombinant sFRP5, an extracellular inhibitor of non‐canonical Wnt signalling, could alleviate cardiac inflammation and protect the heart from ischaemia/reperfusion injury through inhibiting the non‐canonical Wnt5a/JNK signalling and the expression of inflammatory cytokine/chemokine in macrophages and ischaemic myocardium.^91^ Likewise, Wnt5a is highly expressed in ischaemic muscles and ECs from mice overexpressing glutaredoxin‐1, an oxidation‐promoting enzyme which is increased in T2DM patients, and exogenous Wnt5a treatment could inhibit the revascularization in hind limb ischaemia via activating the non‐canonical Wnt pathway,^92^ indicating a deleterious role of Wnt5a in diabetic foot. Although the increase in serum concentration of Wnt5a and a negative correlation between serum Wnt5a and glomerular filtration rate are detected in patients with DP,^93^ more studies tend to prove the protective role of Wnt5a in the development of DP. It has been shown that a weak Wnt5a expression is detected in glomerular mesangium of diabetic rat and glomerular cells cultured in high glucose condition. Therefore, curcumin and exogenous superoxide dismutase administration as well as nitric oxide (NO) donor treatment could significantly alleviate the apoptosis of glomerular cells and diabetic renal fibrosis by restoring the canonical Wnt5a pathway.^94^, ^95^, ^96^ Ando *et al* also showed that Wnt5a induced renal AQP2 expression via the activation of the non‐canonical Wnt5a/Ca^2+^ pathway could increase the urine concentration of mice with heritable nephrogenic diabetes insipidus (NDI), a hereditary disease characterized by defective urine concentration ability in kidney.^97^ Taken together, these findings suggest the complicated roles of Wnt5a in the development of T2DM and related complications in a tissue‐specific manner.

Wnt5b shares 78% amino acid sequence identity with Wnt5a, and its expression is detectable in pancreas, adipose and liver. Wnt5b expression is up‐regulated in islets of mice fed with high‐fat diet (HFD), and UK Caucasian individuals carrying IVS3C>G variant (rs2270031) in the *Wnt5b* gene are predispose to T2DM.^58^, ^98^ Although the exact role of Wnt5b in the regulation of β‐cell proliferation and functions has not been elucidated, silencing the non‐canonical Wnt pathways induced by Wnt4/Wnt5a/Wnt5b and canonical Wnt3a/β‐catenin signalling together could drive the immature hiPSC‐derived S7 cells towards a mature phenotype, indicating the potential inhibitory effect of Wnt5b on the maturation of β‐cells.^48^ Subsequent in vitro experiments showed that compared with the inhibitory effect on adipogenesis via activation of canonical Wnt signalling mediated by Wnt1 and Wnt10b, Wnt5b was overexpressed in 3T3‐L1 cells at an early phase of adipogenesis and could stimulate adipogenesis by antagonizing the canonical Wnt pathway,^99^, ^100^ suggesting that Wnt5b may contribute to the susceptibility to T2DM; therefore, down‐regulation of non‐canonical Wnt5b pathway could therefore decrease adipogenesis and increase β‐cell functions of T2DM subjects.

### 3.5 Wnt6

Wnt6 is highly homologous to Wnt1 but these two Wnts only share 43% amino acid sequence identity. The expression level of Wnt6 is decreased during adipogenesis and ectopic expression of Wnt6 suppresses the differentiation of 3T3‐L1 pre‐adipocytes through a β‐catenin‐dependent pathway,^101^ and activation of Hedgehog signalling has been shown to prevent HFD‐induced obesity in mice by inducing the canonical Wnt6 pathway in adipose tissues.^102^ Thus, induction of Wnt6 could ameliorate metabolic abnormalities such as obesity and T2DM. Moreover, Wnt6 is expressed in mesonephros of the developing mouse embryo, and loss of Wnt6 is obvious in tubulointerstitium of patients with DN and animal models with renal fibrosis; therefore, activating the canonical Wnt6 pathway suppresses renal fibrosis through inhibiting TGF‐β1‐induced activation of NF‐κB pathway.^103^ The activation of ATF3/NFAT axis causes podocyte injury during the development of DN, and ATF3 is increased in glomeruli from proteinuric patients with DP, and Wnt6 is up‐regulated in ATF3‐overexpressed podocytes and identified as a target of this axis, indicating Wnt6 may aggravate podocyte injury and loss.^104^ Furthermore, the activation of Wnt6/β‐catenin pathway in diabetic context establishes a pathological link between T2DM and cancer due to the inducing effect of this signalling on the amplification of centrosome, a symbolic event associated with high‐grade tumours and poor prognosis.^105^ Thus, further studies are needed to explore the role of Wnt6 in the development of T2DM and related complications.

### 3.6 Wnt7a and Wnt7b

Wnt7a and Wnt7b are also secreted proteins with 78% amino acid sequence identity, and both can activate the canonical and non‐canonical Wnt pathways. Although their expression levels have not been determined in islets of diabetic patients, some studies showed that Wnt7a and Wnt7b are required for pancreatic development through autocrine and paracrine mechanisms^106^, ^107^; therefore, stably expressing Wnt7a or Wnt7b alone could enhance the proliferation of human pancreatic progenitor cells (PPCs) through activating the non‐canonical Wnt/PKC signalling, suggesting their promising application in developing cell therapies for diabetes.^107^ Moreover, the expression level of Wnt7a is decreased significantly in cultured human umbilical vein endothelial cells (HUVECs) treated with high glucose and in wounded skin tissues from diabetic rat; thus, localized injection of Wnt7a could reverse the overwhelmed inflammation in wounded skins and accelerate the wound healing rate of diabetic rat.^108^ Similarly, dietary supplementation with resveratrol enhances the expression of hippocampal Wnt7a and neurogenesis of diabetic mice, indicating a potential neuroprotective role of Wnt7a in diabetes.^109^ Moreover, Wnt7b has dual effect during diabetic arteriosclerosis. Wnt7b is detected in aortic tissues and essential for stabilizing normal phenotype and integrity of aortic endothelial cells (AoECs); therefore, specific deletion of Wnt7b in AoECs induces the arteriosclerotic injury in LDLR knockout mice fed diabetogenic diets.^110^ However, the expression of Wnt7a and induced canonical pathway is also induced in aortic tissues from diabetic mice exhibiting cardiovascular calcification; therefore, suppressing vascular calcification could inhibit the aortic mRNA level of Wnt7a.^52^, ^111^ In general, most studies tend to prove that Wnt7a and Wnt7b are potential therapeutic candidates to ameliorate T2DM and related complications.

### 3.7 Wnt8a, Wnt8b, Wnt9a and Wnt9b

Wnt8a and Wnt8b share 63% amino acid sequence identity. At present, little is known about their role in the development of diabetes, and only one study indirectly showed that chimeras between Xenopus Wnt8 and mouse Fzd1 or Fzd2 could inhibit adipogenesis through the canonical and non‐canonical Wnt pathways.^112^ Wnt9, formerly named Wnt14, shares 63% amino acid sequence identity with its analogue Wnt9b. Wnt9a is expressed in embryonic pancreas but not necessary for the formation and growth of pancreas; Wnt9a ablation inhibits the canonical Wnt pathway and leads to the up‐regulation of some genes in endocrine differentiation programme but increase in pancreatic endocrine cell number, including α‐cells, β‐cells and δ‐cells, supporting its negative regulation on endocrine differentiation.^113^ However, there are few studies regarding the role of Wnt9b in the development of T2DM and related complications. Rundqvist et al found a positive correlation between Wnt9a expression and insulin sensitivity, and sprint exercise markedly induced the expression of Wnt9a in human skeletal muscles and up‐regulated the secretion of plasma insulin.^114^ Wnt9b may play a deleterious role in the development of diabetic foot ulcers, because inhibiting the canonical Wnt9b pathway by circulating exosomal miR‐20b‐5p from T2DM patients is found to suppress the angiogenenic effect of HUVECs in vitro and delay the wound healing in vivo.^115^

### 3.8 Wnt10a and Wnt10b

Wnt10a is another biphasic Wnt ligand that shares 62% amino acid sequence identity with Wnt10b. Although the exact role of Wnt10a in diabetes remains unclear, it is indeed important for adipogenesis and several studies have revealed their potential connection.^116^ Wnt10a inhibits the pre‐adipocyte‐adipocyte transition, and the expression of Wnt10a and mediated canonical Wnt pathway is induced in brown pre‐adipocyte cells that show decreased ability to differentiate.^117^ In another study, disruption of circadian clocks in mice results in increased adipogenesis and obesity through silencing the canonical Wnt10a pathway, indicating the protective role of Wnt10a in the development of obesity.^118^ However, Wnt10a and mediated canonical Wnt signalling are also induced in spinal cord of diabetic rat, and dexmedetomidine treatment could alleviate diabetic neuropathy pain by inhibiting the canonical Wnt10a pathway.^119^ The role of Wnt10b in the development of T2DM is comparatively well understood. Among all Wnts, Wnt10b is important negative regulator of adipocyte differentiation via activating the canonical Wnt pathway; lower Wnt10b expression and down‐regulation of Wnt10b/β‐catenin pathway are detected in white adipose and skeletal muscle tissues from men with overweight and prediabetes,^120^ and inactivation of Wnt10b/β‐catenin pathway promotes the adipogenesis and diet‐induced obesity in mice.^121^ On the contrary, transgenic mice in which Wnt10b is overexpressed in adipose resist body fat accumulation and glucose intolerance when fed with HFD or on ob/ob background.^122^, ^123^ Similarly, ectopic expression of Wnt10b in skeletal muscles decreases adipose deposits, hyperinsulinaemia and triglyceride plasma levels and improves glucose homeostasis in adult diet‐induced obese rats.^124^ Activation of Wnt10b/β‐catenin pathway also increases the insulin sensitivity of skeletal muscle cells by decreasing lipid deposition in myoblasts through down‐regulation of SREBP‐1c^125^ and participates in curcumin‐induced suppression of adipocyte differentiation.^126^ Moreover, the expression of Wnt10b is inhibited in bone of mice with T1DM, and elevation of TNF‐α is found to be a critical factor leading to its down‐regulation and bone loss in diabetic environment; thus, overexpression of Wnt10b in bone has a potential utility for the treatment of bone loss in diabetic patients.^127^ These findings demonstrate that Wnt10b blocks the development and function of adipose tissues and improves the glucose homeostasis and insulin sensitivity of whole body.

### 3.9 Wnt11

Wnt11 shows no homology to other Wnts and only shares 41% amino acid sequence identity with Wnt4. Wnt11 is expressed at low level throughout the development; it is mainly expressed in mesenchymal cells of the pancreas but weakly in the areas of endocrine cells.^128^ Studies on adiponectin transgenic mice showing higher sensitivity to insulin revealed that hyperadiponectinaemia resulted in the down‐regulation of Wnt11 expression and chronic inflammation in adipose tissue, but the increase in the number of small adipocyte, a type of ‘good’ adipocyte.^129^ Consistently, high levels of glucose selectively enhanced the expression of Wnt11 in mesenchymal progenitor cells (MPCs) to stimulate adipogenesis through the non‐canonical Wnt/PCP pathway.^130^ These findings suggest that Wnt11 in adipose tissue contributes to the development of obesity‐linked disorders including T2DM.

### 3.10 Wnt16

Wnt16 is another Wnt ligand having two distinct mRNA isoforms, Wnt16a and Wnt16b, which only differ in the composition of 5’‐untranslational region and one exon. Wnt16b is expressed ubiquitously at significant levels in most adult tissues, whereas Wnt16a is barely expressed with high levels in pancreas.^131^ Therefore, most studies on Wnt16 mainly refer to Wnt16b, and it is known to be a major determinant of bone homeostasis and fracture susceptibility in humans. Interestingly, osteoblast‐derived Wnt16 only activates the non‐canonical Wnt pathway in osteoclast progenitors to inhibit osteoclastogenesis, but activates both canonical and non‐canonical Wnt pathways in osteoblasts to increase osteoprotegerin expression and decrease osteoclastogenesis; mice with targeted deletion of Wnt16 in osteoblasts will develop spontaneous fractures due to low cortical bone thickness.^132^ Diabetic patients have higher risk of fracture; the reduction in Wnt16 expression and mediated canonical Wnt signalling activity in cortical bone in diabetic environment might be responsible for the osteopenia, and activating Wnt16/β‐catenin pathway improves the bone strength and provides a therapeutic opportunity for the treatment of osteopenia in diabetic patients.^133^ Moreover, the expression of Wnt16 is decreased in the penises of mice with diabetic erectile dysfunction and overexpression of Wnt16 accelerated the tube formation in cultured mouse cavernous ECs, suggesting the up‐regulation of Wnt16 might contribute to the treatment of diabetic‐related erectile dysfunction.^134^ However, further studies are still required to clarify the role of Wnt16 in the development of diabetes.

## 4 ROLES OF MAIN ANTAGONISTS AND CORECEPTOR OF WNTS IN T2DM AND RELATED COMPLICATIONS

The signalling transduction of Wnt pathways will be blocked if their receptors are bound by competitive antagonists. Secreted frizzled‐related proteins (sFRPs) and Wnt inhibitory factor‐1 (WIF‐1) are classical Wnt antagonists that block all Wnt signalling pathways. As a family of soluble glycoproteins with five members containing cysteine‐rich domain (CRD) homologous to Fzd, sFRPs inhibit all Wnt signalling pathways by competing with Wnt ligands for binding Fzd and play important roles in the pathogenesis of T2DM and related complications. Human foetal aorta‐derived CD133+ progenitor cells accelerate the wound healing of diabetic ischaemic ulcers by activating Wnt pathways, whereas the presence of sFRP‐1 could abolish the reparative process by reducing CD133 expression in progenitor cells. Moreover, the expression of sFRP1, sFRP3 and sFRP4 is up‐regulated when foetal CD133(+) cell differentiation into CD133(−) cells,^135^ indicating the deleterious role of sFRPs in diabetic wounds. However, inhibiting the expression of sFRP‐1 by miR‐27a could aggravate diabetic nephropathy by activating the canonical Wnt signalling.^136^ In human subjects, circulatory sFRP2 is increased in patients with impaired glucose tolerance (IGT) and promotes the adipose angiogenesis through enhancing VEGF expression.^137^ sFRP3 levels in serum and skeletal muscles are significantly reduced in T2DM patients and positively correlated with insulin sensitivity; thus, the treatment of myotubes with recombinant sFRP3 could significantly restore the inhibited insulin signalling induced by cytokine.^138^ The gene expression of sFRP4 is increased in adipose tissues from obese individuals and T2DM patients and in islets from T2DM patients. Circulatory sFRP4 levels are also increased in patients with IGT and T2DM. Moreover, sFRP4 expression is positively correlated with the NAFLD activity score, elevated HbA1c level and insulin resistance, and reduced GSIS, suggesting that elevated SFRP4 is a valuable biomarker of β‐cell dysfunction, insulin resistance and T2DM.^139^, ^140^, ^141^ Therefore, systemically elevated sFRP4 not only inhibits insulin secretion and GSIS in pancreatic β‐cells,^139^ but induces the insulin resistance and lipogenesis in the liver.^141^ sFRP5 is an anti‐inflammatory adipokine that exerts a promising therapeutic effect on inflammatory diseases, including obesity and T2DM, via antagonizing the non‐canonical Wnt5a signalling pathway, and the exact roles of sFRP5 in the pathogenesis of these diseases have been systematically outlined by Shen et al.^142^

The inhibitory effect of WIF‐1 on Wnt pathways is exerted by its directly binding to Wnt ligands. However, there is little evidence on the role of WIF‐1 in the pathogenesis of T2DM and related complications. It is reported that the circulatory WIF‐1 and sFRP‐1 levels were significantly higher in non‐diabetic subjects who developed cardiovascular disease during the follow‐up period, suggesting the elevation in WIF‐1 may be a valuable predictor for future cardiovascular events.^143^ The expression of WIF‐1 is also much higher in diabetic rat induced by STZ treatment. Jinmaitong (JMT), a compound prescription of traditional Chinese medicine, was found to ameliorate DPN in rat via relieving the inhibitory effect of WIF‐1 on the canonical Wnt pathway.^144^ In short, the general effect of WIF‐1 seems to be deleterious diabetic environment.

LRP5/6 are essential coreceptors for the signalling transduction of the canonical Wnt signalling, whereas LRP5 is less effective than LRP6 for the activation of the Wnt pathway. Here, we only discuss the role of LRP6 in the pathogenesis of T2DM and related complications due to the limited space. LRP6 is required for the normal expression of insulin receptor and the stability of IGF receptor in humans, and a rare missense mutation in Wnt coreceptor LRP6 (R611C) is the aetiology of the autosomal dominant early‐onset coronary artery disease, T2DM and metabolic syndrome through blocking the canonical Wnt signalling.^145^, ^146^, ^147^ The homozygote LRP6 knockout is lethal for mice, whereas the heterozygote LRP6 knockdown mice are resistant to glucose intolerance and obesity on HFD; this is associated with the inhibition in the canonical Wnt pathway and resultant increase in mitochondrial biogenesis and reduce in endogenous hepatic glucose output.^148^ Moreover, the concentration of LRP6 is higher in the vitreous samples from patients with proliferative DR,^149^ indicating the deleterious role of LRP6 in diabetes. However, reduced LRP6 expression and inactivation of canonical Wnt pathway are found to promote the progression of DN via enhancing GSK3β‐p53 interaction and the apoptosis of podocyte, and the deleterious effect can be prevented by green tea.^150^ Interestingly, Towler and colleagues found that vascular smooth muscle LRP6 could inhibit the progression of diabetic arteriosclerosis in mice by restraining a novel non‐canonical Wnt/USF1 signalling, indicating a protective role of LRP6 in diabetes.^151^ Therefore, the exact role of LRP6 in the pathogenesis of T2DM and related complications remains an intriguing question and needs in‐depth study.

## 5 OPPORTUNITIES AND CHALLENGES IN DEVELOPING THERAPEUTICS FOR T2DM AND COMPLICATIONS BASED ON WNTS

As mentioned above, there is a close relationship between the dysregulation of Wnt signalling pathways and development of T2DM and related complications. Therefore, appropriate therapeutic approaches targeting Wnt signalling pathways are attractive for the treatment of these disorders, and some reagents that activate or inhibit Wnt signalling pathways have been developed and are undergoing pre‐clinical and clinical trials. For related information described in detail, please refer to review by Aamir et al^152^; herein, we only discuss main challenges in developing therapeutics for T2DM and related complications based on Wnts.

It remains elusive concerning mechanisms that control the generation of distinct Wnts from a single *Wnt* gene. It may be attributed to different promoters regulating the expression of *Wnt* gene and regulators controlling the post‐translational modification, and both of which are simultaneously regulated by a wider range of other signalling pathways. Recently, sFRPs are found to act as the molecular switch to repress or promote specific non‐canonical Wnt signalling branches. Kaufmann and colleagues revealed that the presence of sFRP2 in the extracellular space could activate the non‐canonical Wnt5a/Ror2 signalling by stabilizing Wnt5a‐Ror2 complexes, but reduce the non‐canonical Wnt5a signalling mediated by Fzd7, a main receptor that activates the non‐canonical Wnt/PCP signalling, by preventing Fz7 endocytosis.^153^ Moreover, Wnt signalling pathways are complicated networks in which different Wnts bind to various receptors and activate different effector pathways in a highly dose‐specific and tissue‐specific manner; thus, canonical and non‐canonical Wnt pathways activated by different Wnts may counteract with each other by different concentrations or in different tissues. Interestingly, the interaction of different Wnts could even change the activity of different Wnt signalling pathways. For instant, despite being the biphasic Wnt ligands, Wnt5a and Wnt11 mainly activate the non‐canonical Wnt signalling pathway when secreted alone. However, the post‐translational O‐sulphation of specific tyrosine residues in Wnt11 and Wnt5a will facilitate their interaction and enhance the canonical signalling activity that is required for the initiation of embryonic development.^154^ Therefore, it is not surprising that we observe contradiction in the role of Wnts during the development of T2DM and complications, or even opposite effect of a specific Wnt on different tissues in similar diabetic environment. However, there is increasing evidence that Wnt signalling pathways have certain function in common. In brief, canonical Wnt pathway mediated by most Wnts plays a protective role in alleviating β‐cell dysfunction, excessive adipogenesis in obesity, insulin resistance and T2DM. Thus, activation of canonical Wnt pathway has potential therapeutic effect on obesity and T2DM, and several Wnt activators, such as genistein, kirenol, curcumin and isoquercitrin, have shown beneficial effects on obesity and T2DM at pre‐clinical level via activating the canonical Wnt pathway.^152^ However, most of these activators are targeting components in canonical Wnt pathway rather than Wnts, and their efficacy and safety are only evaluated in cells or diseased animal models rather than in clinical trials. Furthermore, activation of canonical Wnt pathway may accelerate the progression of some diabetic complications, and diabetic patients have an increased risk of cancers due to the direct effect of hyperglycaemia and indirect effects of insulin resistance, hyperinsulinaemia and chronic inflammation,^155^, ^156^ and overactivation of canonical Wnt pathway serves as primary determinant for most human malignancies; hence, systemic application of Wnt activators on diabetic patients may aggravate certain diabetic complications or increase the risk of cancer incidence.

Compared with the conflicting role of canonical Wnt pathway, non‐canonical Wnt pathways are consistently overactivated in diabetes and related complications, and the expression of related Wnts, including Wnt4, Wnt5a, Wnt5b and Wnt11, is increased in diseased tissues in diabetic environment. Therefore, inhibition of the non‐canonical Wnt pathway is a promising therapeutic approach for the treatment obesity, T2DM and related complications. Post‐translational lipidation of Wnts is indispensable for their secretion and activity; almost all Wnts are modified with palmitic acid at conserved serine residue by porcupine (Porcn), a membrane‐bound O‐acyltransferase,^157^ and several small molecule inhibitors targeting Porcn have been developed to prevent Wnts‐driven diseases. LGK974, a Porcn inhibitor, has neuroprotective potential and could ameliorate DPN in rats.^158^ However, our unpublished data showed that treating diabetic mice with another Porcn inhibitor, C59, could exacerbate DPN by activating some crucial inflammatory pathways induced by the silence of canonical Wnt pathway. Consistently, C59 blocks the progression of mammary cancer in mice via down‐regulating the canonical Wnt1/β‐catenin pathway.^159^ We predict the conflicting results might be attributed to the heterogenous function and balance of canonical and non‐canonical pathways during different time windows. Importantly, the normal signalling transduction of Wnt pathways is essential for the maintenance of tissue homeostasis and regeneration; therefore, systemic application of Wnt inhibitors may inhibit some protective Wnts and has unwanted side effects or even toxic on normal tissues when treating diabetic‐related diseases. Therefore, overexpressing Wnts that are aberrantly decreased or inhibiting overexpressed Wnts in determined tissues by using tissue‐specific delivery systems may be viable for treating these disorders. Indeed, several Wnt‐neutralizing antibodies and decoy receptors for Wnts have been developed and promising pre‐clinical trail results have been obtained in treating some human malignancies,^160^ and there is an urgent need to evaluate their efficacy and safety in treating diabetic‐related diseases at clinical level.

## 6 CONCLUSIONS

The dysregulation of Wnt signalling pathways is important pathogenetic basis of series of human diseases, including T2DM and related complications. In recent decades, we have witnessed the remarkable progress in understanding of Wnt signalling pathways in these diseases on a mechanistic level. The function of non‐canonical Wnt pathways activated by few Wnts is consistent in diabetic‐related diseases, while the canonical Wnt pathway activated by most Wnts is either protective or deleterious in a context‐dependent manner, and it is impossible to produce therapeutic effect on these diseases via silencing or activating Wnt pathways alone. Therefore, it is required to have a comprehensive understanding of activation mechanisms and interactions of canonical and non‐canonical Wnt pathways in specific tissues and time windows during the occurrence and progression of diabetic‐related diseases. In this review, we systematically summarize the existing findings on the role of all human Wnts, their main antagonists (sFRPs and WIF‐1) and coreceptor (LRP6) in the development of T2DM and related complications and discus current main challenges in designing novel therapeutic strategies targeting Wnts for the treatment of these disorders. We believe a comprehensive understanding of their functions may pave the way for Wnts to serve as promising therapeutic targets for the prevention and treatment of diabetic‐related diseases.

## CONFLICT OF INTEREST

The authors have no conflicts of interest to disclose in relation to this review.

## AUTHOR CONTRIBUTIONS

**xiaobo Nie:** Conceptualization (lead); Funding acquisition (equal); Project administration (lead); Supervision (lead); Writing‐original draft (lead); Writing‐review & editing (lead). **Xiaoyun Wei:** Formal analysis (supporting); Visualization (supporting); Writing‐original draft (supporting). **Han Ma:** Validation (equal); Writing‐review & editing (equal). **Lili Fan:** Validation (equal); Writing‐review & editing (equal). **Wei‐Dong Chen:** Conceptualization (equal); Funding acquisition (equal); Supervision (equal).
